# Biliary Duct Hamartomas: A Systematic Review

**DOI:** 10.7759/cureus.25361

**Published:** 2022-05-26

**Authors:** Abdul Ahad E Sheikh, Anthony P Nguyen, Katarina Leyba, Nismat Javed, Sana Shah, Alexander Deradke, Christopher Cormier, Rahul Shekhar, Abu Baker Sheikh

**Affiliations:** 1 Internal Medicine, The Wright Center for Graduate Medical Education, Scranton, USA; 2 Internal Medicine, University of New Mexico School of Medicine, Albuquerque, USA; 3 Internal Medicine, Shifa International Hospital, Islamabad, PAK; 4 Medicine, Aga Khan University, Karachi, PAK; 5 Pathology, University of New Mexico School of Medicine, Albuquerque, USA

**Keywords:** multiple biliary duct hamartomas, biliary duct hamartomas, multiple biliary hamartomas, von meyenburg complexes, biliary hamartomas

## Abstract

Biliary duct hamartomas are benign intrahepatic bile duct lesions. Despite being primarily incidental findings on imaging, these lesions can provide a diagnostic conundrum due to their shared characteristics with malignant tumors. The goal of this systematic review is to offer a thorough clinical profile of biliary duct hamartomas.

There were 139 cases of biliary duct hamartomas identified in a structured systematic review of the literature. Patient demographics, clinical presentation, significant laboratory and imaging data, diagnostic modalities, treatment choices, and outcomes were all studied and reported.

Biliary duct hamartomas present with mild symptoms and laboratory abnormalities, and while being visible on imaging, the results are non-specific and may require biopsy in case of red flag signs such as weight loss and a progressive increase in the size of the lesion. Furthermore, there are currently no published guidelines for the treatment of biliary duct hamartomas, and many people have had surgery despite the clinically benign nature of these abnormalities. As per the findings of the study, individuals who exhibit signs of malignancy should be investigated further. Eyeballing for red flag symptoms, followed by a specialized imaging scan and invasive treatment, is the three-step approach to biliary duct hamartomas. Since our recommendations include a shift in strategy and do not contradict existing rules, there are likely to be few roadblocks to improvement; the key barriers being technological equipment and image quality.

In this study, we intended to pave the way for future research in the field. In our opinion, the next decade will bring a better understanding of the characteristics of biliary hamartomas, disease symptoms, and better recognition of any suspicious features. These indications will aid in reducing the number of unneeded surgical or invasive operations. Finally, the findings of these future studies will allow the medical community to improve and provide the best care possible.

## Introduction and background

Biliary duct hamartomas, often known as 'von Meyenberg complexes,' are benign bile duct malformations [1,2]. These clinically benign lesions are frequently discovered accidentally during imaging or laparotomy and might resemble malignant lesions by presenting with clinical symptoms that may necessitate a lengthy diagnostic workup and even surgical intervention. Because of its rarity and diverse clinical presentation, management of this condition can be challenging [1,3]. As a result, detecting and distinguishing biliary hamartomas from other diseases is critical.

Biliary duct hamartomas are malformations of the tiny interlobular bile ducts caused by the failure of embryonic bile duct involution. Their estimated frequency on autopsy ranges from 0.6% to 5.6% and is around 1% on imaging [4, 5]. Although biliary duct hamartomas have been observed in children, they are most common in adults over the age of 35 [1]. Furthermore, polycystic liver and polycystic kidney disease patients are much more likely to have them [6]. They are also more common in individuals with cirrhosis, indicating that environmental exposure plays an important role in their pathophysiology [1,7].

The majority of biliary hamartomas are asymptomatic, however, they might cause fever, jaundice, or abdominal pain. Similarly, even though most patients have a normal clinical exam and lab findings, rare abnormalities in transaminases, alkaline phosphatase, or gamma-glutamyl transferase (GGT) levels have been reported [1]. Even while their clinical history is often benign, as is the case with other ductal plate malformation diseases, biliary hamartomas do pose a minor risk of malignant transition to intrahepatic cholangiocarcinoma or, less commonly, hepatocellular carcinomas [8]. Given the significant variations in care of biliary duct hamartomas and other disorders with similar symptoms, it is critical to adequately define them to aid in correct diagnosis and effective management. Our systematic review seeks to provide a comprehensive clinical picture of biliary duct hamartomas, as well as an up-to-date summary of all cases published in the literature, to identify any prominent and distinctive characteristics.

This article was previously posted to the medRxiv preprint server on April 30, 2021 [9].

## Review

Methods

Protocol Development and Systematic Review Registration

We developed the protocol for this review per the Preferred Reporting Items for Systematic Reviews and Meta-Analyses (PRISMA) criteria after obtaining consensus with all of the reviewers and topic experts. Following protocol preparation and the International Prospective Register of Systematic Reviews (PROSPERO) registration (CRD42021230745), the data search was carried out as described below.

**Figure 1:**
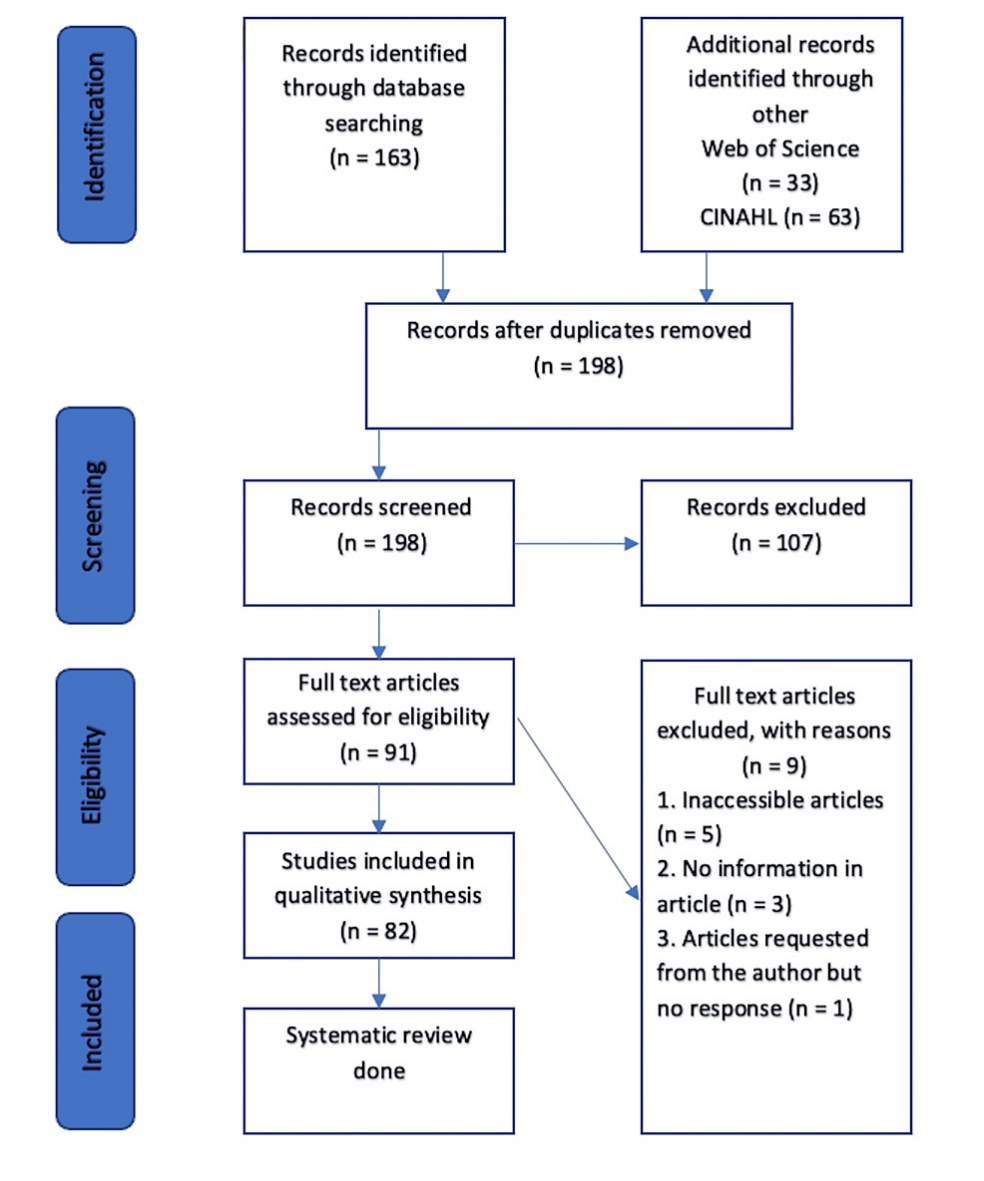
PRISMA flow chart PRISMA: Preferred Reporting Items for Systematic Reviews and Meta-Analyses, CINAHL: Cumulated Index to Nursing and Allied Health Literature

Search Strategy

The following keywords were used in the search strategy: "Biliary hamartomas" OR "Von Meyenburg Complexes" OR "Biliary duct hamartomas" OR "Hamartomas AND biliary" OR "Multiple biliary hamartomas" OR "Multiple biliary duct hamartomas."

Data Extraction (Selection and Coding)

PubMed, Web of Science, and Cumulated Index to Nursing and Allied (CINAHL) were used to conduct our search. This systematic review includes articles published in English during the last thirty years. During the initial search, 163 articles were found on PubMed, 33 on Web of Science (excluding Medline), and 63 on CINHAL (excluding Medline). Following the first search, we deleted all duplicates and imported all included studies into the EndNote (Thomson Reuters, Toronto) online software (Figure 1). The remaining studies were reviewed by two separate reviewers based on the inclusion criteria. The researchers were blinded to each other's findings to avoid bias. Mendeley desktop and Rayyan applications were utilized. The screening process began with the abstracts and progressed to the full-text articles if needed. The initial review included publications published in English as well as those with English translations. Following the first screening, the full-text articles were reviewed for final inclusion by two independent reviewers. The reviewers were blind to each other's conclusions, and a third reviewer was called in to resolve any potential disagreements.

Following that, information on the study's design and methodology, participant demographics and baseline characteristics, the country where it was conducted, the published journal, clinical presentation, laboratory and imaging data, interventions, treatment, clinical outcomes, morbidity, and mortality were extracted from the study records.

One reviewer extracted data, while another double-checked the collected information for accuracy and completeness. Any discrepancies between individual judgments were handled by the third reviewer. Through email correspondence with investigators, we attempted to retrieve any missing data from the research. On a case-by-case basis, studies were removed from the analysis if data could not be collected. This analysis also omitted papers that had not been peer-reviewed. The preliminary data was recorded in an excel spreadsheet, and the final analysis contained 82 studies.

Risk of Bias (Quality) Assessment

The methodological quality and synthesis of case series and case reports as stated by Murad et al. (2018) [10] were used to assess the quality of all included studies (see Tables 1, 2).

**Table 1:** Tool used to evaluate the methodological quality of included case reports and case series

Leading explanatory questions	The question used in the evaluation
1. Does the patient(s) represent(s) the whole experience of the investigator (Centre) or is the selection method unclear to the extent that other patients with a similar presentation may not have been reported?	Yes
2. Was the exposure adequately ascertained?	Yes
3. Was the outcome adequately ascertained?	Yes
4. Were other alternative causes that may explain the observation ruled out?	Yes
5. Was there a challenge and/or rechallenge phenomenon?	No
6. Was there a dose-response effect?	No
7. Was follow-up long enough for outcomes to occur?	Yes
8. Is the case(s) described with sufficient details to allow other investigators to replicate the research or to allow practitioners to make inferences related to their practice?	Yes

**Table 2:** Quality assessment of the included studies (n=82)

Quality assessment of the included studies				
Judgment	%	N	Case study (n= 67)	Case series (n=15)
Good	90.2	74	59	15
Fair	9.8	8	8	0
Poor	0.0	0	0	0

According to this technique, four broad viewpoints were employed to assess quality: study group selection, ascertainment of the observed outcome, causation of the observed outcome, and case reporting.

Strategy for Data Synthesis

We utilized Statistical Package for the Social Sciences (SPSS) version 21 (IBM Corp., Armonk, NY, USA) for statistical analysis. Continuous data were expressed as mean ± standard deviation based on the distribution of data, whereas qualitative variables were expressed as frequency and percentages.

Results

There have been 139 cases of bile duct hamartomas published in the literature to date, comprising 68 case reports and 15 case series. Our analysis includes these 139 patients. Tables 3, 4, 5 [2,7,8,11-90] summarise the data from all of the cases studied.

**Table 3:** Demographic characteristics of the cases reviewed NA: Not available, M: Male, F: Female

Article	Age	Sex	Comorbidities			Time to presentation
			Hepatitis B	Hepatitis C	Cancer/Others	
Andres et al. (2006) [11]	50	M	-	-	-	NA
Barboi et al. (2013) [2]	71	M	-	-	-	NA
Beard et al. (2014) [12]	48	F	-	Positive	-	NA
Bieze et al. (2013) [13]	44	F	-	-	-	NA
Brunner et al. (1994) [14]	65	F	-	-	-	NA
Del Nero et al. (2015) [15]	65	M	-	-	-	NA
Dilli et al. (2012) [16]	61	F	-	-	-	NA
Fritz et al. (2006) [17]	66 & 47	2M	-	-	Esophageal carcinoma x2	NA
Fuks et al. (2009) [18]	56	F	-	-	Breast cancer	10 years
Gil Bello et al. (2012) [19]	56	M	-	-	Prostate cancer	NA
Gong et al. (2015) [20]	57	M	-	-	-	Several years
Guiu et al. (2009) [21]	53	F	-	-	Breast cancer	NA
Gupta et al. (2016) [22]	53	M	-	-	-	1 month
Gupta et al. (2017) [23]	33	M	-	-	-	NA
Hasebe et al. (1994) [24]	59	M	-	-	Sigmoid colon cancer	NA
Hashimoto et al. (2011) [25]	75	M	-	-	-	NA
Heinke et al. (2008) [26]	19 & 39	1F & 1M	-	-	-	NA
Jain et al. (2000) [27]	63, 81 & 66	2M & 1F	-	Positive in 81-year-old male	Breast cancer in a 66-year-old female	NA
Jain et al. (2010) [28]	55 to 60	4 M	-	Positive x2	-	NA
Jang et al. (2014) [29]	44	F	-	-	Caroli's disease	NA
Jáquez-Quintana et al. (2017) [30]	50	M	-	-	-	NA
JF Blanc et al. (2000) [31]	61	M	-	-	-	NA
JF Krahn et al. (2012) [32]	52	F	-	-	-	NA
Kim et al. (2011) [33]	66	M	-	-	-	NA
Kim et al. (1999) [34]	74	M	-	-	-	NA
Koay et al. (2016) [35]	62	M	-	-	-	7 years
Kobayashi et al. (2004) [36]	30	M	-	-	-	NA
Li et al. (2009) [37]	71	F	Positive	-	-	NA
Li et al. (2020) [38]	40	M	-	-	-	NA
Lin et al. (2018) [39]	62 & 57	2F	Positive x2	-	-	NA
Lin et al. (2013) [7]	17 to 63	4M & 2F	Positive x1	-	-	NA
Liu et al. (2005) [40]	61	M	-	-	-	NA
Liu et al. (2014) [41]	28-66	5M & 7F	Positive x3	-	Gastric cancer x2, Colorectal cancer x3, Thyroid cancer x1, Lung cancer x1	NA
Lung et al. (2013) [42]	81	M	-	-	-	1 week
Madakshira et al. (2019) [43]	50	M	-	-	-	NA
Madhusudhan et al. (2009) [44]	61	M	-	-	-	1 month
Madigan et al. (2015) [45]	45	M	-	-	Polycystic liver disease	NA
Manjunath et al. (1999) [46]	57	M	-	-	-	3 months
Mansour et al. (2011) [47]	51	F	-	-	-	NA
Merkley et al. (2005) [48]	71	F	-	-	-	5 years
Michalakis et al. (2011) [49]	54	M	-	-	-	NA
Mimatsu et al. (2008) [50]	60	M	-	-	-	1 year
Miura et al. (2007) [51]	55	M	-	-	-	1 week
Nagano et al. (2006) [52]	76	M	-	-	Common bile duct cancer	NA
Nasr et al. (2006) [53]	45	M	-	-	-	NA
Neri et al. (2004) [54]	51	M	-	-	-	NA
Neto et al. (2006) [55]	70	F	-	Positive	-	3 weeks
Neubert et al. (2020) [56]	52	F	-	-	-	3 months
Panaro et al. (2004) [57]	55	M	-	-	-	NA
Panda et al. (2019) [58]	62	F	-	-	-	NA
Parekh et al. (2013) [59]	76	F	-	-	-	NA
Park et al. (2007) [60]	53	M	-	-	-	NA
Pinho et al. (2012) [61]	56	F	-	-	-	2 months
Rocken et al. (2020) [62]	59	F	-	-	-	NA
Saidi et al. (2013) [63]	49	F	-	-	-	NA
Sato et al. (2012) [64]	59	M	-	-	-	1 year
Sato et al. (2009) [65]	63	M	-	-	-	NA
Schlachterman et al. (2015) [66]	45	M	-	-	-	NA
Sharma et al. (2016) [67]	73	M	-	-	-	NA
Shi et al. (2015) [68]	37	M	Positive	-	-	6 months
Shin et al. (2011) [69]	62 & 79	1F & 1M	-	-	Colon cancer in a 79-year-old male	NA
Shirazi et al. (2019) [70]	65	M	-	-	-	1 month
Sinakos et al. (2011) [71]	25-60	2M & 2F	-	-	-	3 days to 6 months
Singh et al. (2017) [72]	55	M	-	-	-	NA
Song et al. (2008) [8]	59-75	4M	Positive x1	Positive x1	Fibrocystic liver disease x1	NA
Sugawara et al. (2008) [73]	55	M	-	-	-	3 years
Suzuki et al. (2008) [74]	56	M	-	-	Advanced sigmoid colon cancer	NA
Swinnen et al. (1995) [75]	63	F	-	-	-	NA
Tarar et al. (2015) [76]	57	M	-	-	-	1 week
Teng et al. (2015) [77]	73	M	-	-	-	2 days
Tohme et al. (2008) [78]	33-68	4M & 7F	-	-	Gastric carcinoma x1	NA
Turdean et al. (2011) [79]	66	M	-	-	-	NA
van Baardewijk et al. (2010) [80]	74	F	-	-	-	NA
Van Dorpe et al. (2017) [81]	38	F	-	-	-	NA
Varotti et al. (2017) [82]	63	M	NA	NA	NA	NA
Vitule et al. (2010) [83]	27	1F	-	-	-	2 days
Waledziak et al. (2018) [84]	58	1M	-	-	-	NA
Xu et al. (2009) [85]	88 & 65	1M & 1F	-	Positive in a 65-year-old female	-	NA
Yang et al. (2017) [86]	35-70	7M & 2F	Positive x5	-	-	NA
Yoh et al. (2004) [87]	69	M	-	-	-	NA
Yoshida et al. (2009) [88]	88	F	-	-	-	NA
Zheng et al. (2005) [89]	29 to 72	4M & 2F	-	-	-	NA
Zimpfer et al. (2007) [90]	73	M	-	-	-	NA

**Table 4:** Pathological findings of cases reviewed +: Positive, -: Negative, CK7: Cytokeratin 7, NA: Not available, MRCP: Magnetic resonance cholangiopancreatography, H&E: Hematoxylin and eosin, HTN: Hypertension, w/: With, WI: Weighted imaging, AFP: Alpha-fetoprotein, CA 19-9: Carbohydrate antigen 19-9, CK19: Cytokeratin 19, CK20: Cytokeratin 20, p53: Tumor protein p53, PAS: Periodic acid-Schiff, w/o: Without, TTF-1: Thyroid transcription factor 1, p63: Tumor protein 63, CEA: Carcinoembryonic antigen, HCC: Hepatocellular carcinoma

Article	Tumor Markers	MRI and Biopsy	Diagnosis (Number of Lesions)	Management	Outcome
Andres et al. (2017) [11]	NA	MRI: Multiple hypointense diffuse liver lesions of variable size, Biopsy: NA	Biliary hamartoma (multiple lesions)	NA	Alive
Barboi et al. (2013) [2]	Normal	MRI: Multiple small cystic lesions were detected with T1 hyposignal and T2 hypersignal, the largest being in segment 7, Biopsy: NA	Biliary hamartoma (multiple lesions)	Supportive	Alive
Beard et al. (2014) [12]	NA	MRI: Tubulocystic composition and intermingled normal hepatic tissue, Biopsy: Thick, dense fibrous tissue containing cytologically bland, large-caliber bile ducts with intermingled benign hepatocytes	Biliary adenofibroma in setting of biliary hamartomas (multiple lesions)	Surgical	Alive
Bieze et al. (2013) [13]	Normal	MRI and Biopsy: NA	Biliary hamartoma (multiple lesions)	Surgical	Alive
Brunner et al. (1994) [14]	Normal	MRI: 2 hypointense cysts in the 4th and 8th hepatic segments, Biopsy: Regular epithelium in the setting of dense fibrous stroma, pericentrolobular cholestasis	Biliary hamartoma without malignancy (multiple lesions)	Surgical	Alive
Del Nero et al. (2015) [15]	Normal	MRI: Numerous small (<1.0 to 1.5 cm diameter) liver lesions, hypointense on T1-weighted scans and hyperintense on heavily T2-weighted scans, not communicating with the bile ducts, Biopsy: NA	Biliary hamartoma (multiple lesions)	NA	Alive
Dilli et al. (2012) [16]	Normal	MRI: Multiple, small, nodular lesions with slightly irregular contours in the liver parenchyma, measuring 1.5 cm or less in size. The lesions were hypointense on T1-weighted images and hyperintense on T2-weighted images, Biopsy: NA	Biliary hamartoma (multiple lesions)	Supportive	Alive
Fritz et al. (2006) [17]	NA	MRI and Biopsy: NA x2	Biliary hamartoma x2 (multiple lesions)	Surgical x2	Alive
Fuks et al. (2009) [18]	Normal	MRI: Multiple and small lesions with high signal intensity scattered throughout the liver, especially in the subcapsular area in T2-weighted images. After contrast injection, the multiple masses showed irregular progressive enhancement from the arterial phase through a delayed phase, Biopsy: Fibrosis	Biliary hamartoma (multiple lesions)	Surgical	Alive
Gil Bello et al. (2012) [19]	Normal	MRI: NA, Biopsy: Multiple nodules composed of multiple bile ducts; periductal fibrous stroma and fibrous bridges between nodules	Biliary hamartoma (multiple lesions)	Autopsy finding	Death
Gong et al. (2015) [20]	Normal	MRI: Multiple small cysts of hypointense on T1-weighted images and hyperintense on T2-weighted images, Biopsy: NA	Biliary hamartoma (multiple lesions)	NA	NA
Guiu et al. (2009) [21]	Normal	MRI: Multiple irregularly delineated lesions hyperintense and cystic, less than 1 cm, Biopsy: Biliary epithelium with fibrous stroma	Biliary hamartoma (multiple lesions)	NA	NA
Gupta et al. (2016) [22]	Normal	MRI: Dilated bilateral intrahepatic bile duct radicles with a collapsed gallbladder. There was a presence of a 2.5 by 2.0 cm heterogeneous lesion with altered intensity at the porta. The common bile duct was not visualized, Biopsy: Multiple biliary hamartomas with complete ductal plate dysgenesis	Biliary hamartoma with Klatskin tumor (multiple)	Surgical	Alive
Gupta et al. (2017) [23]	Normal	MRI and Biopsy: NA	Biliary hamartoma	NA	Alive
Hasebe et al. (1994) [24]	Elevated CEA	MRI: NA, Biopsy: Site 1 - Bile duct adenoma and atypical ductules growing into the liver parenchyma, granular/hyperchromatic large nuclei. Site 2 - Metastatic adenocarcinoma from sigmoid colon	Bile duct adenocarcinoma with a focal area of biliary hamartoma	Surgical	Alive
Hashimoto et al. (2011) [25]	Normal	MRI and Biopsy: NA	Biliary hamartoma	Supportive	Alive
Heinke et al.(2008) [26]	Normal	MRI: NA, Biopsy: Patient 1 - Well-differentiated pattern with polygonal cells, vesicular nuclei, prominent nucleoli, irregularly dilated bile ducts w/ fibrous stroma consistent with bile duct hamartomas. Patient 2 - 3.2cm tumor w/ central fibrosis consistent with HCC and biliary hamartomas	Hepatocellular carcinoma with underlying biliary hamartomas x2	Autopsy finding x1; surgical x1	Death x1; alive x1
Jain et al. (2000) [27]	Normal x3	MRI: NA, Biopsy: Diffuse nodularity and fibrosis, numerous biliary hamartomas, ductal proliferations showing adenocarcinoma, neoplastic changes in biliary hamartoma (irregular contours, inssipated bile, hyalinized stroma) x3	Intrahepatic cholangiocarcinoma x3	Surgical x1; autopsy finding x1; NA x1	Death x2; NA x1
Kobayashi et al. (2004) [36]	NA	MRI: NA, Biopsy: Discrete, periportal cysts embedded in a fibrous stroma, lined by a low columnar or cuboidal epithelium and contained bile-stained material, some dilated	Biliary hamartoma	Surgical	Alive
Li et al. (2009) [37]	Normal	MRI and Biopsy: NA	Cholangiocarcinoma arising from an underlying biliary hamartoma	Surgical	Alive
Li et al. (2020) [38]	NA	MRI: Rounded, irregular, low-signal-T1 and high-signal-T2 lesions diffusely distributed on the liver which were not significantly enhanced, Biopsy: NA	Biliary hamartoma	Supportive	Alive
Lin et al. (2018) [39]	NA	MRI: NA, Biopsy: Patient 1 - Biliary hamartoma and changes consistent with chronic hepatitis B. Patient 2 - NA	Patient 1: Hepatocellular carcinoma & biliary hamartoma; Patient 2: Biliary hamartoma	Supportive x1; NA x1	Alive
Liu et al. (2005) [40]	Normal	MRI: 4 by 3.7 cm well-defined lesion, with low signal intensities on T1-weighted images and high signal intensities in the central portion on T2-weighted images. Biopsy: Expanded portal areas, with variably dilated and disorganized bile ducts, embedded in the fibrous stroma, and containing altered bile. No evidence of malignancy.	Biliary hamartoma (Multiple lesions)	Supportive	Alive
Liu et al. (2014) [41]	Normal	MRI: Hypointense x12, hyperintense x12, rim-like enhancement x6; Biopsy: Lesions were subsequently classified into class 1 (predominantly solid lesions with narrow bile channels), class 2 (intermediate, with mild or focal dilatation of bile channels), and class 3 (predominantly cystic). Class 1: 0, Class 2: 8, Class 3: 4	Biliary hamartomas x 12	NA x12	Alive x12
Lung et al. (2013) [42]	Normal	MRI: NA, Biopsy: Normal background hepatic parenchyma. Focal lesions: expanded portal tracts with dense stroma, containing dilated bile ducts; a few of which contained inspissated bile. One dilated congested blood vessel.	Biliary hamartoma	Surgical	Alive
Madakshira et al. (2019) [43]	Normal	MRI: NA, Biopsy: Bile-stained lesions, likely micro-hamartomas composed of a collection of dilated varying-sized ducts lined by cuboidal epithelium in a fibrotic stroma. The cells lining these ducts were positive for CK7, confirming them to be of bile duct origin	Biliary hamartoma	Autopsy finding	Death
Madhusudhan et al.(2009) [44]	Normal	MRI: Diffusely scattered lesions, less than 1 cm in size, in both lobes of the liver, which were hypointense on the T1-weighted image and hyperintense on the T2-weighted image, Biopsy: NA	Biliary hamartoma (multiple lesions)	Surgical	Alive
Madigan et al. (2015) [45]	Normal	MRI: NA, Biopsy: Fibrosis, hepatocellular extinction, mild iron deposition	Polycystic liver disease and biliary hamartoma	NA	Alive
Manjunath et al. (1999) [46]	Normal	MRI: NA, Biopsy: Dilated bile ducts lined by attenuated bland epithelium. Inspissated bile seen in one duct	Biliary hamartoma	Supportive	Alive
Mansour et al. (2011) [47]	Normal	MRI: NA, Biopsy: Irregularly shaped ductular structures lined by cuboidal epithelium, surrounded by dense fibrous stroma (interface between the biliary hamartoma and the liver parenchyma)	Biliary hamartoma	Supportive	Alive
Merkley et al. (2005) [48]	Elevated CA-A 19	MRI: ]Multiple small cysts throughout the liver and a 1.0 × 1.5 cm focus in the left lobe, Biopsy: Biliary hamartoma	Biliary hamartoma (multiple lesions)	NA	Alive
Michalakis et al. (2011) [49]	Normal	MRI: NA, Biopsy: Intraportal proliferations of small to medium-sized irregular ductuli lined by benign flattened biliary epithelium, embedded within fibrous stroma	Biliary hamartoma	NA	Alive
Mimatsu et al. (2008) [50]	Normal	MRI: NA, Biopsy: Multiple dilated bile ducts with surrounding fibrous stroma	Biliary hamartoma	Surgical	Alive
Miura et al. (2007) [51]	Normal	MRI: NA, Biopsy: Aggregation of irregular-shaped cystic dilated bile ducts embedded in fibrous stroma, with minimal inflammatory reaction	Biliary hamartoma (multiple lesions)	NA	Alive
Nagano et al. (2006) [52]	Normal	MRI: Multiple irregularly delineated hyperintense nodules were seen, not communicating with the biliary tree, Biopsy: Multiple bile ducts with slightly dilated lumens embedded in the collagenous stroma	Biliary hamartoma (multiple lesions)	Surgical	Alive
Nasr et al. (2006) [53]	Normal	MRI: NA, Biopsy: Biliary hamartoma	Biliary hamartoma x2	Surgical x1; supportive x1	Alive
Neri et al. (2004) [54]	Normal	MRI: NA, Biopsy: Periportal cysts on the mono‐stratified cubical epithelium and a conspicuous increase in connective tissue	Biliary hamartoma	Supportive	Alive
Neto et al. (2006) [55]	Normal	MRI: Well-circumscribed mass within segments 2 and 3 of the liver, Biopsy: Tumor within the lumen of the bile duct without invasion into the liver parenchyma	Intraductal papillary cholangiocarcinoma (single lesion)	Surgical	Alive
Neubert et al. (2020) [56]	Normal	MRI: Multiple innumerable cysts, Biopsy: NA	Biliary hamartoma (multiple lesions)	Surgical	Alive
Panaro et al. (2004) [57]	Normal	MRI: NA, Biopsy: Dilated bile ducts lined by cuboidal epithelium that were embedded in a dense fibrous stroma	Biliary hamartoma	Surgical	Alive
Panda et al. (2019) [58]	Normal	MRI: Multiple innumerable lesions, Biopsy: Biliary hamartoma without evidence of advanced fibrosis	Biliary hamartoma (multiple lesions)	NA	Alive
Parekh et al. (2013) [59]	Normal	MRI: NA, Biopsy: Well-differentiated cholangiocarcinoma and numerous biliary hamartomas. Tumor cells were positive for CK7 and CA19-9 with focal staining for CK20, but negative for TTF-1 and p63	Cholangiocarcinoma	Surgical	Alive
Park et al. (2007) [60]	Normal	MRI and Biopsy: NA	Biliary hamartoma	NA	Alive
Pinho et al. (2012) [61]	Normal	MRI: NA, Biopsy: Bile duct adenoma	Intrahepatic cholangiocarcinoma in the setting of bile duct adenomas	Surgical	Alive
Rocken et al. (2000) [62]	Normal	MRI: NA, Biopsy: Non-encapsulated tumor with columnar/cuboidal cells with necrosis, likely poorly differentiated adenocarcinoma. Liver nodules scattered throughout were found to be biliary hamartomas	Intrahepatic cholangiocarcinoma	Surgical	Alive
Saidi et al. (2013) [63]	Normal	MRI and Biopsy: NA	Biliary hamartomas	NA	Alive
Sato et al. (2009) [64]	Normal	MRI: Multiple small hyperintense nodules that were scattered throughout the liver but did not communicate with the biliary tree, Biopsy: NA	Biliary hamartoma (Mutiple lesions)	NA	Alive
Sato et al. (2012) [65]	Normal	MRI: NA, Biopsy: Hepatic cysts lined by cuboidal, flattened biliary‐type epithelium without atypia	Biliary hamartoma	NA	Alive
Schlachterman et al. (2015) [66]	Normal	MRI: Extensive multinodularity, Biopsy: Biliary hamartomas and features of ductal plate malformation	Biliary hamartomas	NA	Alive
Sharma et al. (2016) [67]	Normal	MRI and Biopsy: NA	Biliary hamartomas	NA	Alive
Shi et al. (2015) [68]	NA	MRI: Round lesion measuring 15 mm in diameter was found in the upper segment of the right anterior hepatic lobe, with hypo-intense signals on T1-weighted images, and hyper-intense signals on T2-weighted images (T2WI), and high diffusion on diffusion-weighted imaging (DWI), respectively. Rim enhancement, Biopsy: Irregularly-arranged small bile ducts surrounded by fibrocollagenous stroma. The bile duct epithelia were significantly hyperplastic without cellular heteromorphism	Biliary hamartoma (multiple lesions)	Supportive	Alive
Shin et al. (2011) [69]	Normal	MRI: NA, Biopsy: Patient 1 - Biliary hamartoma with cystic dilation, Patient 2 - NA	Biliary hamartoma	NA x1; surgical x1	Alive
Shirazi et al. (2019) [70]	Normal	MRI- Low signal intensity on T1-weighted image, and high signal intensities on T2-weighted image and diffusion image. After contrast enhancement, it showed a subtle rim-like enhancement. Biopsy: Many proliferating bile ducts are lined by a single layer of uniform cuboidal epithelium without mitotic activity. Mild-to-moderate fibrosis around these ductules.	Biliary hamartoma (single lesion)	Supportive	Alive
Sinakos et al. (2011) [71]	NA x2, Normal x2	MRI: Presence of multiple cystic liver lesions with a maximum size of 1.5cm. These lesions had no communication with the bile ducts, Biopsy: NA	Biliary hamartoma (multiple lesions)	NA x2; supportive x2	Alive
Singh et al. (2017) [72]	Normal	MRI: NA, Biopsy: Cyst wall composed of loose collagenous tissue lined by a single layer of cuboidal cells in keeping with biliary type epithelium. The adjacent liver parenchyma contained several ectactic bile ducts with focal branching. The surrounding stroma was densely hyalinized with a mild lymphocytic infiltrate and dilated lymphatic channels	Biliary hamartoma and cyst (multiple lesions)	Surgical	Alive
Song et al. (2008) [8]	Elevated CA-A 19 x1, NA x3	MRI: Low signal intensity in T1-weighted imaging, Biopsy: Tumor composed of benign biliary hamartomas characterized by irregular and dilated ducts without luminal bile. Multiple foci of adenocarcinoma consisting of infiltrative and dysplastic glandular/solid structures. Dysplasia shown as pseudostratification comprising cells with enlarged and variably sized nuclei and some apical snouts x3; fibrocystic liver disease x1	Metastatic colon cancer with biliary hamartoma x2; presumed unusual type of hepatocellular carcinoma or hemangioma with biliary hamartoma x1; hepatic adenocarcinoma with biliary hamartoma x1	Surgical x4	Death x1; alive x3
Sugawara et al. (2008) [73]	Elevated CA-A 19	MRI: Hypointense on T1, hyperintense on T2 x1, Biopsy: Intrahepatic cholangiocarcinoma with diffuse microhamartomas and dilated/irregular hyperplastic bile ducts	Cholangiocarcinoma with concurrent biliary hamartoma	Surgical	Alive
Suzuki et al. (2008) [74]	Normal	MRI: Multiple hypointense and hyperintense hepatic nodules on T1-weighted gradient echo images and T2-weighted fast spin echo images, respectively. On heavily T2-weighted images, the signal intensity increased. Resovist-enhanced T1-weighted image showed no enhancement of these lesions, Biopsy: NA	Biliary hamartoma (multiple lesions)	Supportive	Alive
Swinnen et al. (1995) [75]	Normal	MRI: NA, Biopsy: Multiple, tortuous, dilated bile ducts, lined with normal cuboidal epithelium and embedded in a fibrous stroma	Biliary hamartoma	Supportive	Alive
Tarar et al. (2015) [76]	Normal	MRI and Biopsy: NA	Biliary hamartoma	Supportive	Alive
Teng et al. (2015) [77]	Normal	MRI: Tiny lesions ranging from 1 mm to 10 mm diffusely distributed in both lobes. These lesions had low signal intensity on T1-weighted images and increased signal intensity on T2-weighted images. The signal intensity onT2-weighted images were slightly less than that of simple fluid. Biopsy: Multiple biliary channels lined by the regular cuboidal epithelium with dense fibrous stroma	Biliary hamartoma (Multiple lesions)	Supportive	Alive
Tohme et al. (2008) [78]	Normal	MRI: All lesions were hypointense on T1 and hyperintense on T2-weighed images, Biopsy: Numerous preserved biliary hamartomas sustained by hyalinized collagenous stroma were observed inside the tumor, some of them were malignant.	Biliary hamartoma x9; cholangiocarcinoma x1	NA	Alive x11
Turdean et al. (2011) [79]	Normal	MRI: NA, Biopsy: Bile ducts were lined with simple columnar epithelium, focally flattened, without nuclear atypia. The luminal content was amorphous, slightly eosinophilic, and negative to the Alcian blue-PAS stain. The stroma between these ducts was dense, rich in collagen fibers, and was continued by the stroma of adjacent portal areas. A reduced quantity of interstitial Alcian blue-positive mucin was also present	Biliary hamartoma	Surgical	Alive
van Baardewijk et al. (2010) [80]	Normal	MRI: NA, Biopsy: Biliary hamartomas	Biliary hamartoma	NA	Alive
Van Dorpe et al. (2017) [81]	Normal	MRI: Multiple small liver lesions hyperintense on T2-weighted images and hypointense on T1-weighted images with no enhancement after intravenous administration of gadolinium, Biopsy: NA	Biliary hamartomas	NA	Alive
Varotti et al. (2017) [82]	Normal	MRI and Biopsy: NA	Biliary hamartoma	Surgical	Alive
Vitule et al. (2010) [83]	Normal	MRI: NA, Biopsy: Cystic dilations involving granulomatous fibrotic tissue of intrahepatic biliary ducts	Biliary hamartomas	Surgical	Alive
Waledziak et al. (2018) [84]	Normal	MRI: NA, Biopsy: Initial resected tumor - Low-grade intrahepatic cholangiocarcinoma without vascular invasion, CK7+, CK19+, CK20-, p53+, PAS-, CK20-. Subsequent right hepatectomy: focal bile duct hamartoma w/o signs of malignancy	Low-grade intrahepatic cholangiocarcinoma	Surgical	Alive
Xu et al. (2009) [85]	Elevated AFP and CA-A19 x1, Normal x1	MRI: NA, Biopsy: Tumor with areas of dysplastic ductal cells consistent with adenocarcinoma that appear to transition from mild dysplasia to invasive, inflammatory infiltrates with lymph follicles in connective tissue, necrosis present x2; cavitation lesions x1	Intrahepatic cholangiocarcinoma x2	Surgical x2	Death x2
Yang et al. (2017) [86]	Normal	MRI: 5/8 patients revealed hypointensity on T1-weighted imaging (WI) and hyperintensity on T2WI. A total of 4 patients exhibited enhancement and 1 exhibited no enhancement in the arterial phase, Biopsy: Bile duct proliferation x6; Bile duct proliferation, partly nest-like arrangement x1; Bile duct proliferation, partly nest-like arrangement, liver cirrhosis x1; Obvious atypical cells, invasive growth, partly bile duct proliferation, cholangiocarcinoma x1	Biliary hamartomas x6; Biliary duct hamartomas with partial malignant transformation x3	NA	Alive x8
Yoh et al. (2004) [87]	Normal	MRI: T1-weighted images revealed a low-density mass, T2-weighted images revealed a dappled-density mass w/ honeycomb-like dilated distal bile duct and dilation of the major intrahepatic bile duct, Biopsy: Multicystic mass consisting of dilated cystic bile ducts, periductal glands, connective tissue, and blood vessels with thickened walls. Findings consistent with biliary hamartoma, suggestive of multicystic biliary hamartoma	Multicystic biliary hamartoma (multiple lesions)	Surgical	Alive
Yoshida et al. (2009) [88]	Normal	MRI: Multiple small hyperintense lesions <1 cm diameter and normal intrahepatic and extrahepatic bile ducts. Inferior vena cavography and hepatic venography showed no angiostenosis or thrombus in the major blood vessels. Biopsy: Diffuse bile duct hamartomas. H&E staining showed bile duct microhamartomas consisting of circumscribed fibrous areas containing many irregularly dilated bile duct structures and only a few narrowed vessels in the portal region.	Biliary hamartoma and portal HTN (Multiple lesions)	Supportive	Alive
Zheng et al. (2005) [89]	Normal	MRI: Multiple small hepatic lesions were demonstrated to be of low signal intensity on T1-weighed images and high signal intensity on T2-weighed images with no enhancement (3/6). MRCP clearly portrayed numerous tiny irregular-shaped and few round-shaped small hyper-intense nodules (2/3), Biopsy: Multiple dilated bile ducts lined by a single layer of cubic epithelium x2	Biliary hamartoma x5	NA	Alive x5
Zimpfer et al. (2007) [90]	Normal	MRI (NA), Biopsy: Multiple dilated bile ducts, some containing bile plugs, small glandular units lined by cuboidal cells with marked atypia, dense desmoplastic stroma that invaded the liver parenchyma. CK7+	Intrahepatic cholangiocarcinoma and biliary hamartoma	NA	NA

**Table 5:** Clinical presentation and laboratory findings of the cases reviewed AST: Aspartate transaminase, ALT: Alanine transaminase, GGT: Gamma-glutamyl transferase, NA: Not available, RUQ: Right upper quadrant, LUQ: Left upper quadrant

Article (Year)	Symptoms			Labs			Clinical exam
	Abdominal pain	Jaundice	Fever	AST/ALT	GGT	Bilirubin	
Andres et al. (2017) [11]	-	-	-	Elevated	Elevated	-	NA
Barboi et al. (2013) [2]	-	-	-	-	-	-	Hepatomegaly
Beard et al. (2014) [12]	Present	-	-	-	-	-	NA
Bieze et al. (2013) [13]	Present	-	-	Elevated	-	-	NA
Brunner et al. (1994) [14]	-	-	-	-	-	-	NA
Del Nero et al. (2015) [15]	-	-	-	-	Elevated	-	Hepatomegaly
Dilli et al. (2012) [16]	Present	-	-	-	-	-	NA
Fritz et al. (2006) [17]	-	-	-	NA	NA	NA	NA
Fuks et al. (2009) [18]	-	-	-	NA	NA	NA	NA
Gil Bello et al. (2012) [19]	-	-	-	-	-	-	NA
Gong et al. (2015) [20]	Present	-	-	Elevated ALT only	-	Elevated	NA
Guiu et al. (2009) [21]	-	-	-	-	-	-	NA
Gupta et al. (2016) [22]	-	Present	-	Elevated	Elevated	Elevated	Jaundice, hepatomegaly
Gupta et al. (2017) [23]	Present	-	-	-	-	-	NA
Hasebe et al. (1994) [24]	-	-	-	-	-	-	NA
Hashimoto et al. (2011) [25]	-	-	Present	Elevated	Elevated	-	NA
Heinke et al. (2008) [26]	-	Present	-	-	-	-	NA
Jain et al. (2000) [27]	Present x2	-	-	Elevated ALT only x1	-	-	Splenomegaly x2, ascites x2, varices x1, portal HTN x1
Jain et al. (2010) [28]	Present x2	-	Present x1	-	-	-	NA
Jang et al. (2014) [29]	-	-	-	-	-	Elevated	NA
Jáquez-Quintana et al. (2017) [30]	-	-	-	Elevated AST only	Elevated	-	NA
JF Blanc et al. (2000) [31]	-	-	-	-	Elevated	-	NA
JF Krahn et al. (2012) [32]	-	-	-	-	Elevated	-	HTN
Kim et al. (2011) [33]	-	-	-	-	Elevated	-	NA
Kim et al. (1999) [34]	-	-	-	-	-	-	NA
Koay et al. (2016) [35]	Present	Present	Present	NA	NA	NA	Positive Murphy’s sign, tenderness in RUQ
Kobayashi et al. (2004) [36]	-	-	-	NA	NA	NA	NA
Li et al. (2009) [37]	-	-	-	-	-	-	NA
Li et al. (2020) [38]	-	-	-	-	-	-	NA
Lin et al (2018) [39]	-	-	-	Elevated x2	-	-	NA
Lin et al (2013) [7]	-	Present x1	-	Elevated x1	-	-	NA
Liu et al. (2005) [40]	-	-	-	-	-	-	NA
Liu et al. (2014) [41]	-	-	-	-	-	-	NA
Lung et al. (2013) [42]	Present	-	-	-	-	-	Abdominal distension and guarding
Madakshira et al. (2019) [43]	-	-	-	-	-	-	NA
Madhusudhan et al. (2009) [44]	-	-	-	-	-	-	NA
Madigan et al. (2015) [45]	-	-	-	-	-	-	Splenomegaly, gynecomastia, +1 pedal edema
Manjunath et al. (1999) [46]	Present	-	-	-	-	-	Hepatomegaly
Mansour et al. (2011) [47]	-	-	-	Elevated ALT only	Elevated	-	NA
Merkley et al. (2005) [48]	Present	-	-	-	-	-	NA
Michalakis et al. (2011) [49]	-	-	-	-	-	-	NA
Mimatsu et al. (2008) [50]	-	-	-	-	-	-	NA
Miura et al. (2007) [51]	Present	Present	-	Elevated	Elevated	Elevated	Jaundice
Nagano et al. (2006) [52]	Present	-	-	-	-	-	NA
Nasr et al. (2006) [53]	Present x1	-	-	-	-	-	Positive Murphy’s sign x1, RUQ pain x1
Neri et al. (2004) [54]	Present	-	-	-	-	-	Hepatomegaly
Neto et al. (2006) [55]	Present	-	-	Elevated	-	-	Positive Murphy’s sign, RUQ tenderness
Neubert et al. (2020) [56]	-	Present	Present	-	-	Elevated	HTN, fever, tachycardia
Panaro et al. (2004) [57]	-	-	-	-	-	-	NA
Panda et al. (2019) [58]	-	-	-	-	-	-	NA
Parekh et al. (2013) [59]	-	-	-	-	-	-	NA
Park et al. (2007) [60]	-	-	-	-	-	-	NA
Pinho et al. (2012) [61]	-	-	-	-	-	-	NA
Rocken et al. (2000) [62]	Present	-	-	-	Elevated	-	NA
Saidi et al. (2013) [63]	-	-	-	Elevated	-	-	NA
Sato et al. (2012) [64]	-	-	Present	Elevated	-	-	Bilateral peripheral edema and distal polyneuropathy
Sato et al. (2009) [65]	Present	-	-	-	-	-	NA
Schlachterman et al. (2015) [66]	-	-	-	Elevated ALT only	Elevated	-	NA
Sharma et al. (2016) [67]	-	-	-	-	-	-	NA
Shi et al. (2015) [68]	-	-	-	NA	NA	NA	NA
Shin et al. (2011) [69]	-	-	-	-	-	-	Weight loss
Shirazi et al. (2019) [70]	Present	-	-	-	-	Elevated	Vague mass in the RUQ
Sinakos et al. (2011) [71]	Present x4	Present x1	Present x2	Elevated x2	Elevated x1	-	RUQ tenderness x2
Singh et al. (2017) [72]	Present	-	-	-	-	-	Vague mass in the LUQ
Song et al. (2008) [8]	-	-	-	NA	NA	NA	NA
Sugawara et al. (2008) [73]	-	-	Present	-	-	-	NA
Suzuki et al. (2008) [74]	-	-	-	-	-	-	NA
Swinnen et al. (1995) [75]	Present	-	-	NA	NA	NA	Normal physical exam
Tarar et al. (2015) [76]	-	-	Present	-	-	-	NA
Teng et al. (2015) [77]	Present	-	Present	-	-	-	Positive Murphy’s sign, RUQ tenderness
Tohme et al. (2008) [78]	-	-	-	-	Elevated	-	NA
Turdean et al. (2011) [79]	Present	-	-	-	-	-	NA
van Baardewijk et al. (2010) [80]	-	-	-	-	-	-	Friable tumor on rectal exam
van Dorpe et al.(2017) [81]	Present	-	-	-	-	-	NA
Varotti et al. (2017) [82]	NA	NA	NA	NA	NA	NA	NA
Vitule et al. (2010) [83]	Present	-	-	-	-	-	NA
Waledziak et al. (2018) [84]	-	-	-	-	-	-	NA
Xu et al. (2009) [85]	Present x2	-	-	Elevated ALT only x1	Elevated x1	-	Positive Murphy’s sign x1, RUQ tenderness x1
Yang et al. (2017) [86]	Present x2	-	-	ALT elevated x2 and AST elevated x3	-	Elevated x2	NA
Yoh et al. (2004) [87]	-	-	-	-	-	-	NA
Yoshida et al. (2009) [88]	-	-	-	-	Elevated	-	NA
Zheng et al. (2005) [89]	-	-	-	-	-	-	NA
Zimpfer et al. (2007) [90]	-	-	-	-	-	-	NA

Patient Demographics

Patients' ages ranged from 17 to 88 years old at the time of diagnosis, with a mean age of 55±13 (mean±SD) years. Males accounted for 63.3% of all reported cases (88 vs 51), suggesting a slight male predominance.

Comorbidities

An underlying comorbidity was identified in 48.9% of the 139 documented cases. Cancer was the most often reported comorbidity, with 12.9% of patients reporting it. Among these, gastrointestinal cancers (esophageal tumors, gastric cancer, common bile duct cancer, and colorectal cancer) were the most prevalent, accounting for 12 out of 18 or 66.7% of all underlying malignancies. Comorbid cancers of the breast, prostate, lung, and thyroid were also reported.

Viral hepatitis was found in 8.6% of the patients in this study, with hepatitis B accounting for seven of the 12 cases (58.3%) and hepatitis C accounting for five of the 12 cases (41.7%). Furthermore, 8.6% of patients had underlying hypertension, and 5% had type 2 diabetes. Tobacco usage (4.3%), alcohol use (3.6%), hemochromatosis (1.4%), and tuberculosis (1.4%) were among the other comorbidities noted.

Clinical Presentation

Only 36% of the patients in this study were symptomatic, whereas the vast majority (almost 2/3 of them) were asymptomatic at the time of presentation. Abdominal pain was the most prevalent presenting symptom among those who were symptomatic, with 22.3% of patients reporting abdominal pain ranging from nonspecific abdominal discomfort to localized epigastric or right hypochondrial pain. Fever (18%), weight loss (14%), and jaundice (14%) were other frequently mentioned symptoms. Clinical symptoms that were less often described were abdominal distension and upper and lower gastrointestinal haemorrhage. The interval between the onset of symptoms and presentation varied amongst studies, ranging from one day to 10 years.

Although clinical exams were recorded inconsistently, a positive Murphys' sign and right hypochondrial soreness to palpation were prevalent among patients who had a physical exam (nine out of 26 or 34.6%), particularly among those who arrived with abdominal pain. Hepatomegaly and splenomegaly were seen in a limited percentage of individuals with documented physical examinations (19.2% and 13.6%, respectively).

Laboratory Findings

Laboratory data, mostly alanine or aspartate transaminases (ALT/AST), were recorded for 66 of the 139 patients in this study. Around 33.3 % of the 66 individuals showed increased transaminase levels to some degree. The levels of gamma-glutamyl transferase (GGT) and alkaline phosphatase (ALP) were measured in 71 individuals, with 42.2 percent (30 of 71) having high GGT or ALP. Bilirubin levels were measured in 60 people in the research, with 15 (25%) having hyperbilirubinemia. Other documented laboratory abnormalities include hypoalbuminemia, increased C-reactive protein (CRP), and hematologic abnormalities such as anaemia, leukocytosis, leukopenia, and thrombocytopenia.

Tumor marker information was recorded for 42 of the 139 patients. The great majority of patients (85.7 percent) had no tumour marker elevations. Carbohydrate antigen 19-9 (CA 19-9), a tumor marker linked with gastrointestinal malignancies, particularly pancreatic malignancies [89,91], was found to be high in four patients (9.5%). One patient (2.4 percent) had high levels of alpha-fetoprotein (AFP), a tumor marker linked to liver and testicular malignancies [91,92]. One patient also had a rise in CEA, a tumor marker related to malignancies in numerous organ systems, including the gastrointestinal system [91].

Imaging

In this analysis, 122 of the 139 patients had some type of abdominal imaging, while the others were diagnosed based on biopsy data. Sixty (43.2%) of the 122 patients had ultrasound imaging, 69 (49.6%) had computed tomography (CT) scans, and 81 (58.2%) had magnetic resonance imaging (MRI) scans. All imaging modalities indicated radiographic abnormalities. The lesions suspected of being benign biliary duct hamartomas were hypoechoic in 50% (30/60) of the ultrasounds, hyperechoic in 21.7% (13/60), and mixed in 28.3% (17/60).

Furthermore, CT scans for suspected lesions indicated hypodensity and hyperdensity in 81.2% (56/69) and 13.0% (9/69) of patients, respectively, whereas 5.8% (4/69) had mixed density lesions. Contrast enhancement was found in 32.5% of the 40 CT scans that included contrast.

This review included studies that used both T1-weighted and T2-weighted MRI images. T1 hypo-intensity was found in 96.3% of the 81 T1-weighted scans for suspected lesions, whereas T1 hyper-intensity was seen in 3.7%. In contrast, T2 hypo-intensity was found in 6.2% of the 80 T2-weighted scans for benign lesions, whereas T2 hyper-intensity was seen in 93.8%. 

Forty-one MRI scans used contrast, with 41.5 % of them reporting contrast enhancement. Several individuals had magnetic resonance cholangiopancreatography (MRCP), which revealed hepatic lesions or cysts that had no relationship to the biliary network. On imaging, however, three of these individuals had dilated biliary ducts, which were discovered by ultrasonography. In one case, the CT scan also indicated dilated biliary ducts. The MRI revealed a similar result in four patients.

Lesion Characteristics

The size and location of the lesions of interest were reported on radiography for 44.6% and 48.2% of the patients, respectively. The average size of the lesions was 1 cm in 53.2% of the cases. Twenty-two of the 67 lesions (32.8%) were uniformly distributed across the liver, whereas 21 of the 67 (31.3%) were limited to a single segment. Only two lesions were on the surface of the liver in the right lobe (16 vs 6). They were frequently diagnosed intraoperatively as grey or white nodular lesions, with some cases indicating a cystic component.

Diagnosis, Management, and Outcome

The majority of the patients in this study had biopsies as part of the diagnostic process. The pathologic diagnosis of benign bile duct hamartoma was found in 69.6% of the 112 instances for which biopsy findings were available. In 8.9% of instances, the pathologic diagnosis was bile duct hamartoma with partial malignant alterations, and in 21.4% of cases, the biopsy material was determined to be consistent with malignancy.

The proportion of patients classified radiologically with a malignancy was comparable to the pathology results when considering the final diagnosis provided in each instance; 26.6% of patients were diagnosed with some sort of malignancy, while 73.3% were determined to have a benign diagnosis.

The management strategies used in each case differed significantly. Around 59% of patients with a recorded treatment method had some sort of surgical intervention, the most prevalent of which was partial hepatectomy. Other individuals had cholecystectomies or liver transplants. Prior to the final procedure, 42.7% of patients that received surgical management got a biopsy. In comparison, 36.1% of patients got supportive treatment. Furthermore, 24.4% of patients who underwent surgical intervention were diagnosed with cancer, the most common of which was cholangiocarcinoma (14/20; 70%). A small proportion of cases (4.8%) had postmortem lesions, with two individuals being diagnosed with cancer (2/7; 28.6%).

Overall, the patients in this study had satisfactory results. Of the 116 instances documented patient outcomes, 107 cases (92.2%) stated that the patients were alive at the time of publication, and nine cases (7.8%), including those describing lesions discovered on autopsy, indicated that the patients were deceased at the time of publication. There were no outcomes in the remaining 23 cases.

Discussion

Our systematic review included 82 studies on the incidence of biliary duct hamartomas. In these studies, 139 people with biliary duct hamartomas presented with symptoms or were identified incidentally on imaging, after surgery, or after a malignant transformation.

Their ages varied from 17 to 88 years, with the average patient age being 55 ±13 (mean±SD) years. In our evaluation, the age range was considerably greater than in another study, which reported biliary duct hamartomas detected in individuals above the age of 35 on average [7]. Male dominance was also discovered (88 male patients vs 51 female patients). This conclusion contrasted with one that found female preponderance for this condition [7].

Approximately half of the patients with malignancies had comorbid conditions, with gastrointestinal malignancies being the most prevalent (12.9 %), followed by chronic viral hepatitis B and C (8.6 %). This study supports earlier findings that biliary hamartomas are not always congenital, but can also be acquired as a result of viral inflammation caused by hepatitis B and C [3]. It's worth noting, however, that this higher incidence might be attributable to selection bias since individuals with chronic liver illness are more likely to receive abdominal imaging.

Multiple investigations [1,89] have found that the majority of the patients were asymptomatic (64%). Abdominal discomfort was the most prevalent symptom reported by patients, followed by fever, weight loss, and jaundice. However, 41 symptomatic patients (82%) were identified with biliary duct hamartomas, whereas nine symptomatic patients (18%) had malignancies (cholangiocarcinoma, hepatocellular carcinoma and metastatic cancer). These symptoms have also been reported in other investigations [91,92].

A third of the 66 patients in our study showed high transaminases, while a quarter had hyperbilirubinemia. These increases can point to two possibilities: derangement in tests might be caused by a big lesion obstructing the biliary network, or the derangement could be caused by transient damage to the lesion [93]. Forty-two out of 139 patients (85.7 %) were found to have normal tumor markers. This was consistent with previously published research [3-6]. However, there were a few cases when CA 19-9, AFP, and CEA levels were increased. This is unlikely to be unique to biliary hamartomas, as increased tumor markers are linked to a variety of cancers and may also be present in situations with underlying liver cirrhosis, portal hypertension, and jaundice [86]. As a result, the significance of tumor markers in biliary duct hamartomas remains debatable.

Ultrasonography revealed a number of common features. The lesions were mostly hypoechogenic (80%) and smaller than 1 cm in diameter (53.2%). This is an intriguing observation, as one research found that smaller lesions are hyperechogenic due to acoustic reflection from tightly apposed walls [41]. As a result, our data support the hypothesis that ultrasonography characteristics for biliary duct hamartomas are usually non-specific [94,95]. On CT scan, the lesions were predominantly hypointense. According to a few studies, these lesions grow clearer rather than enhance with contrast (32.5%), which is a puzzling feature that might lead to a malignancy diagnosis [96,97]. On MRI, the majority of the lesions exhibited hypointense and hyperintense signals on T1 and T2-weighted images, as reported earlier [96,97]. Contrast enhancement was found in 41.5% of the lesions, which might be attributed to the compressed liver parenchyma around the masses [78].

Biliary duct hamartomas were mostly detected on the liver's surface (32.8%), however, they might sometimes be restricted to a segment (31.5%). The right lobe had a greater involvement than the left. It is unknown what causes the differences in lobe participation. However, the diffuse distribution of nodules on the surface of the liver was considered a trait indicative of a benign form of biliary duct hamartomas [96].

The biopsy is the best diagnostic tool and was performed on 112 patients. Despite the fact that the majority of the patients (73.3%) had benign bile duct hamartomas, 59.9% of them underwent some form of surgical procedure as part of their treatment. Surgical therapy may not be necessary until red flag signs, such as weight loss or a rise in the size of the lesion, are observed. Given the benign nature of the lesion, regular surveillance is not indicated in individuals with stable biliary hamartomas. This would save money and eliminate the need for invasive treatments.

As stated in several studies, the results were usually positive, with many patients living long-term unless they had additional illnesses that might potentially worsen their health in the long run [1,93]. This is true for hamartomas in all organ systems; lungs, small bowel, and pancreatic hamartomas have all been documented to have generally positive outcomes [97-99]. Aside from the small chance of malignancy, biliary hamartomas are a benign disorder with no known long-term consequences. As a result, in asymptomatic patients, no intervention is necessary [1].

**Figure 2:**
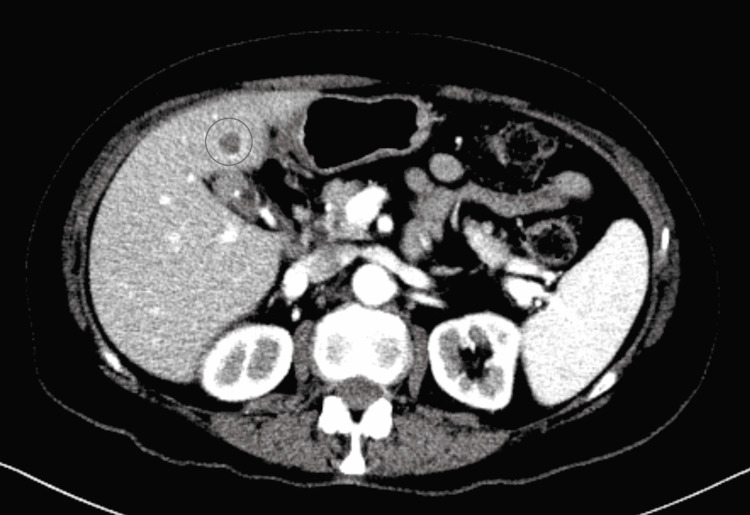
CT of the abdomen and pelvis with contrast showing 1.1 cm hypodense lesion within segment IVb This CT image is used here in the article with written consent from the patient's legal guardian.

**Figure 3:**
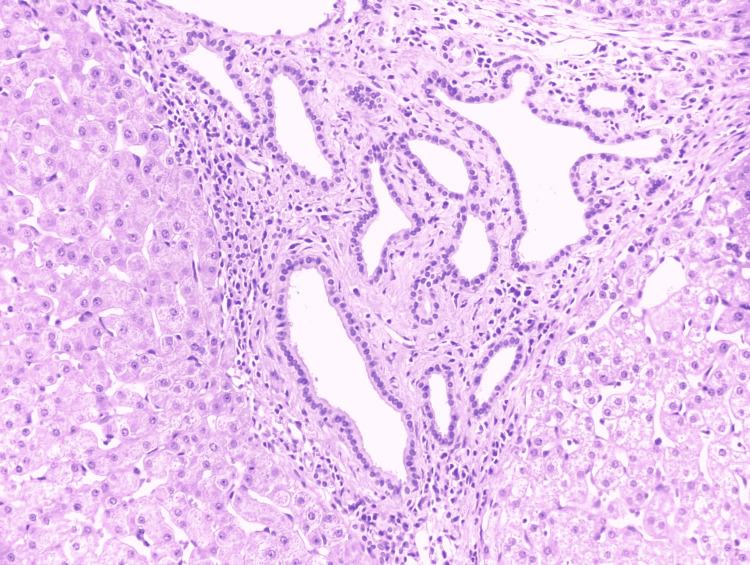
Liver parenchyma showing a well-demarcated lesion with small to medium-sized, irregularly shaped dilated glands lined by bland cuboidal epithelium with intervening fibrous stroma and surrounding inflammatory cells consistent with biliary hamartomas (H&E staining, x20) This image is used here in the article with written consent from the patient's legal guardian. H&E: Hematoxylin and eosin

Bile duct hamartomas are benign lesions that are lined with bile duct epithelium and are frequently dilated due to bile collections in their lumen. The vast majority of cases manifest as non-enhancing, hypoattenuating lesions (Figure 2). The stromal characteristics of these lesions differ, as does the presence of cystic dilatations of intrahepatic bile ducts covered by a single layer of cuboidal cells with fibrous stroma between them (Figure 3). Biliary duct hamartomas pose a diagnostic challenge due to their similar appearance to malignant tumors [3]. As a result, the goal of this study is to give common clinical symptoms, laboratory tests, and imaging results that can help diagnose biliary hamartomas in individuals with atypical hepatic lesions.

The large number of patients allowed for a detailed study of the clinical, diagnostic, and prognostic aspects of biliary duct hamartomas, which is a key strength of this research. One possible drawback is publication bias; instances with substantial or atypical symptoms or those linked with malignant transformation are more likely to be published in the literature than cases with clinically insignificant symptoms.

## Conclusions

Biliary duct hamartomas are benign tumors of the intrahepatic bile ducts that can produce mild symptoms and test abnormalities. However, while symptoms, clinical exam findings, and raised liver enzymes are all valuable markers, they are not specific and may not be diagnostic of biliary duct hamartomas. As a result, imaging methods are critical for diagnosing and treating them. Even though regular monitoring is not indicated due to their benign nature, imaging findings for biliary duct hamartomas are often non-specific, necessitating biopsy, especially in situations with red flag symptoms such as weight loss and a gradual increase in lesion size.

Patients with symptoms suggestive of malignancy should be investigated further, as per the findings of the review. This three-step process of looking for red flag symptoms, a specific imaging scan, and invasive therapy is more of a paradigm change in how bile duct hamartomas are approached. As previously stated, because our recommendations entail a shift in approach rather than contradicting existing norms, there are likely few hurdles to improvement; the key roadblocks are technical equipment and image quality.

There are currently no standard treatment guidelines for biliary duct hamartomas, and even though these malformations are clinically benign, many patients have received surgery. More research is needed in several areas to identify the best strategy to manage this rare disorder, with the ultimate objective of delivering the greatest care to these patients. One of these areas is the development of new and improved guidelines for determining the usage of invasive vs non-invasive approaches to treating biliary duct hamartomas. These guidelines would necessitate additional research, including observational studies, case series, and hospital surveys on imaging, as well as data on imaging modalities. Diagnostic modalities, the kind of modality necessary for benign or malignant lesions in biliary duct hamartomas, and the specificity of each type of modality for the lesions being treated are all factors to take into account. It is crucial to define the features of the lesion more precisely to rule out the possibility of malignancy in biliary duct hamartomas. This distinction would require more precise and clear imaging techniques, which are heavily reliant on the resolution and quality of images generated by various devices. It's possible that in the future, we'll have access to more specific technological advancements that will allow us to better visualize bile duct hamartomas.

This study was done to give insight into the present diagnosis and care of individuals with biliary duct hamartomas while keeping the disease's potential evolution in mind. We also intended to pave the way for further research in this area. In our opinion, the next decade will provide a deeper understanding of biliary hamartomas' characteristics, disease symptoms and processes, and a clearer detection of any suspicious features. Using these cues will drastically reduce the number of unneeded surgical or invasive operations. Ultimately, the findings of all future research will enable the healthcare community to modify and provide the best standards of practice.
